# Investigation of the Effectiveness of First Measured Arterial/Cord Blood Gas and Laboratory Results in Predicting the Treatment Model in Newborns Diagnosed With Transient Tachypnea

**DOI:** 10.1155/jotm/5637693

**Published:** 2026-02-12

**Authors:** Ali Bulbul, Tolga Bacak, Ahmet Yasar Tellioglu, Alper Divarcı, Hasan Avsar

**Affiliations:** ^1^ Division of Neonatology, Department of Pediatrics, Sisli Hamidiye Etfal Education and Research Hospital, University of Health Science, Istanbul, Turkey, sbu.edu.tr

**Keywords:** blood gas, electrolytes, nCPAP, newborn, nSIMV, respiratory support prediction, transient tachypnea of the newborn

## Abstract

**Objective:**

The aim of this study was to investigate the predictive effect of laboratory results and blood gas values on the selection of respiratory support models in infants diagnosed with transient tachypnea of the newborn (TTN).

**Method:**

The study was designed as a single‐center, retrospective study. Infants born with gestational age ≥ 35 weeks diagnosed with TTN during a 2‐year period were included. Demographic characteristics, laboratory parameters, respiratory support models, and length of hospital stay were recorded. The relationship between the obtained parameters and the percentage and duration of oxygen requirement, nasal continuous positive airway pressure (nCPAP), nasal synchronized intermittent mandatory ventilation (nSIMV), intubation, and hospitalization duration was evaluated.

**Results:**

The study was completed with 327 infants. A correlation was found between pH and pCO2 values in the first blood gas analysis and the duration of oxygen administration (*p* : 0.019 and *p* : 0.001), and between serum calcium levels and peak sodium levels and the duration of nSIMV (*p* : 0.04 and *p* : 0.023). Low serum calcium, phosphorus, and initial sodium levels were identified in infants requiring invasive ventilation (*p* : 0.001, *p* : 0.006, and *p* : 0.012, respectively). In the ROC analysis used to predict intubation, the cutoff value for calcium was determined as < 8.11 mg/dL (AUC 0.771, [95% CI: 0.669–0.872], *p* : <0.001). For predicting the need for nCPAP, the cutoff value for pH in the first blood gas analysis was < 7.32 (AUC 0.705, [95% CI: 0.586–0.823], *p* : 0.003), and for predicting the need for nSIMV, the cutoff value for pH was < 7.28 (AUC 0.599, [95% CI: 0.535–0.663], *p* : 0.003).

**Conclusions:**

It was determined that the initial blood gas pH and pCO2 values, as well as serum sodium, calcium, and phosphorus levels, could be used to predict the treatment model in infants diagnosed with TTN. Low calcium, phosphorus, and sodium levels were found in TTN‐diagnosed infants requiring invasive mechanical ventilation.

## 1. Introduction

The most common respiratory problem detected after birth in term and late preterm infants is transient tachypnea of the newborn (TTN). In the early postnatal period, newborn infants must develop many adaptations in the respiratory and circulatory systems to survive postnatally. During the respiratory adaptation process, regular rhythmic breathing, alveolar expansion, and the clearance of fetal lung fluid play a crucial role. Insufficient clearance of fetal lung fluid typically presents as well‐defined tachypnea symptoms within the first 2 h of life. This condition is known as TTN and was first described in 1966 by Avery ME as a delay in the clearance of fetal lung fluid [1]. It has been reported that TTN may lead to hypoxic respiratory failure and persistent pulmonary hypertension in developing infants [2]. Serious complications and associated mortality may rarely be associated with hypoxic respiratory failure or transient pulmonary hypertension. Additionally, TTN is associated with prolonged hospital stays, unnecessary antibiotic use, and separation of the mother–infant pair [2]. Currently, treatment for infants with TTN involves the use of inhaled salbutamol, epinephrine, corticosteroids, diuretics, and/or fluid restriction within the first 48 h of life; however, there are insufficient scientific data to determine the efficacy of these treatments, and none of the current guidelines recommend these therapies [3, 4]. There are very few studies in the current literature addressing TTN and electrolyte imbalance. Several parameters have been evaluated to determine the severity of the disease in infants with TTN. The effectiveness of many parameters, such as blood lactate levels, mean platelet volume (MPV), right ventricular systolic pressure, and reticulocyte count, in determining the prognosis of TTN has been evaluated [5, 6]. Recently, it has been reported that genetic factors play a role in determining the severity of the disease in infants with TTN, and that polymorphisms in genes encoding beta‐adrenergic receptors (ADRB) are associated with the development of the disease [7]. Noninvasive respiratory support models (nCPAP: nasal continuous positive airway pressure, nSIMV: nasal synchronized intermittent mandatory ventilation) are known to play an effective role in TTN treatment [8]. However, there is no accepted standard consensus regarding the superiority of these treatment models over one another.

The aim of our study was to examine the relationship between laboratory and blood gas values in newborns diagnosed with TTN and to evaluate its effectiveness in predicting TTN development and selecting appropriate respiratory support models.

## 2. Method

The study was designed as a single‐center, retrospective cohort study. The files of all infants born at our hospital with a gestational age ≥ 35 weeks during a 2‐year period (between October 2018 and October 2020) were reviewed. Infants diagnosed with TTN at admission were included in the study. Information from the medical records of the included infants, demographic and maternal characteristics, birth details, laboratory parameters, applied respiratory support models, hospitalization duration, and treatments administered, was recorded in the study form. The relationship between laboratory values (blood gas values obtained from umbilical arterial blood or within the first hour after birth from capillary sample, sodium values obtained within the first 12–24 h, blood count values, the highest and lowest sodium values obtained during follow‐up, calcium, phosphorus, and lactate values, thyroid‐stimulating hormone [TSH], and free thyroxine [fT4] levels) was examined in relation to the treatment models applied and their predictive values in forecasting prognosis. All procedures, including blood sampling and decisions regarding the type of respiratory support to be administered, were performed according to our clinic’s protocols. The relationships between laboratory values and the need for oxygen support, nCPAP, nSIMV, intubation, and hospitalization duration were evaluated using linear regression analysis.

The diagnosis of TTN was made in the presence of at least one symptom from the following 3 criteria: 1. presence of signs of respiratory distress (tachypnea > 60/min, moaning, intercostal and subcostal retraction, nasal wing breathing, and cyanosis) starting within the first 6 h after birth; 2. on physical examination, secretory rales or thin rales on auscultation, tachycardia, and mild hepatosplenomegaly; and 3. on chest radiography, perihilar fullness, pulmonary congestion, presence of fluid in interlobar septa and fissures, Kerley A/B lines, minimal pleural effusion, increased ventilation, and widening of intercostal spaces and resolution within 72 h. Respiratory support was initiated in patients with hypoxia (defined as oxygen saturation ≤ 95%) and/or hypercapnia (defined as partial pressure of CO_2_ ≥ 50 mm Hg on room air) [9, 10]. The mode of respiratory support (nCPAP, nSIMV, or intubation) was determined by the treating physician.

## 3. Exclusion Criteria

Infants who were screened for infection after birth due to suspected early neonatal sepsis or congenital pneumonia and who had already started antibiotic treatment were excluded from the study to prevent the accidental inclusion. According to our clinical criteria, infants diagnosed with respiratory distress syndrome or requiring surfactant therapy were excluded from the study. Infants with congenital heart disease (all structural heart anomalies except for physiologically acceptable atrial septal defect) and infants diagnosed with persistent hypoglycemia, hypocalcemia, polycythemia, or meconium aspiration syndrome and infants with major congenital anomalies (e.g., meningomyelocele) or chromosomal anomalies (e.g., Down syndrome) were excluded from the study. Patients with any respiratory diagnosis other than TTN were also removed from the study.

## 4. Ethical Approval

This study was approved by the Ethics Committee of the Clinical Research Center of our hospital on April 27, 2021, with the number 3236/2021.

## 5. Statistical Analysis

Statistical analyses were performed using SPSS Version 20. The normality of variables was examined using the Kolmogorov–Smirnov test and histogram graphs. Descriptive analyses were presented using mean, standard deviation, median, minimum, and maximum values. Categorical variables were compared using Pearson’s and chi‐square tests.

For nonparametric groups, the Mann–Whitney *U* test was used. Spearman’s correlation tests were used to analyze the relationship between measured variables. Linear regression analysis was applied to investigate the factors affecting oxygen administration, nCPAP, nSIMV, intubation, and hospitalization duration. ROC analysis was applied to predict infants who received intubation, nCPAP, and nSIMV and to determine threshold values for parameters that were found to be significant in infants monitored due to TTN. Cases with a *p* value below 0.05 were considered statistically significant.

## 6. Results

During the study period, the files of 3974 patients born at ≥ 35 weeks of gestation in our hospital were retrospectively reviewed. The study was completed with 327 patients who were hospitalized and treated for TTN and whose data were complete. The demographic characteristics of the patients included in the study, the treatment models and durations applied, and the laboratory results are presented in Table 1.

**TABLE 1:** Distribution of demographic characteristics and laboratory results of infants followed up for transient tachypnea of the newborn.

	**Mean ± standard deviation**	**Range**

Gestation age (weeks)	38.6 ± 1.4	35–43
Age of mother (years)	29.6 ± 5.9	17–47
Mode of delivery, cesarean, *n* (%)	236 (72)	
Gender, male, *n* (%)	59 (193)	
Applied respiratory support models	*n* (%)	
Oxygen	327 (100)	
nCPAP	308 (94.2)	
nSIMV	114 (34.9)	
Intubation	31 (9.5)	
Oxygen requirements (days)	2 ± 1.7	1–12
Highest FiO_2_ value (%)	28 ± 7	21–100
Duration of nCPAP (days)	1.1 ± 0.4	1–4
Duration of nSIMV (days)	1.1 ± 0.8	1–6
Duration of intubation (days)	2.6 ± 2.0	1–8

*Frequency of treatments applied*		
Duration of TPN (days) (*n*: 57)	3.4 ± 2.3	1–9
Hospitalization duration (days)	6.2 ± 4.1	1–25

*Laboratory values*		
First measured arterial/cord blood		
pH	7.22 ± 0.18	7.01–7.70
PCO_2_, mmHg	55.3 ± 10.9	22.3–93.8
Serum lactate level, mmol/L	3.3 ± 1.8	0.4–12.4
Calcium, mg/dL	8.5 ± 0.7	6.3–10.3
Phosphorus, mg/dL	6.1 ± 0.9	2.7–9.3
First sodium level, mEq/L	138.4 ± 2.9	125–148
Highest sodium level, mEq/L	141.1 ± 2.8	133–154
Lowest sodium level, mEq/L	136.8 ± 3.2	125–146
TSH, mU/L	5.1 ± 4.6	0.4–38.6
T4, ng/L	15.6 ± 3.4	2–33.6
Albumin, Gr/dL	3.4 ± 0.3	2.5–4.1
Hemoglobin level, Gr/dL	17.6 ± 2	11.6–22
Platelet counts, 10^9^/L	266 ± 63	70–524
MPV, fL	9.2 ± 0.8	7.4–12
PMI	2443 ± 576	616–5135

Abbreviations: MPV, mean platelet volume; nCPAP, nasal continuous positive airway pressure; nSIMV, nasal synchronized intermittent mandatory ventilation; PMI, platelet mass index; TPN, total parenteral nutrition; TSH, thyroid‐stimulating hormone.

The correlation results of respiratory support and laboratory parameters in infants monitored for TTN are presented in Table 2. A positive correlation was found between duration of oxygen therapy and pCO_2_ and the highest sodium values. A negative correlation was observed between duration of oxygen therapy and the first blood gas pH, calcium, phosphorus, lowest sodium, hemoglobin, hematocrit, and albumin values. There was a positive correlation between duration of intubation and TSH levels. A negative correlation was found between duration of intubation and MPV levels. There was a positive correlation between nSIMV duration and the highest sodium level, while a negative correlation was found between nSIMV duration and calcium levels. There was a negative correlation between the hospitalization duration and calcium, phosphorus, lowest sodium, MPV, and albumin levels. A positive correlation was found between the hospitalization duration and the highest sodium level. It was found that a one‐unit increase in pCO_2_ value increases the duration of oxygen therapy by 2.7%. A one‐unit increase in calcium value reduces the duration of oxygen therapy by 0.476 times. It was found that a one‐unit increase in sodium at the highest level increases the duration of oxygen therapy by 0.19 times. A one‐unit increase in sodium at the lowest level decreases the duration of oxygen therapy by 0.161 times (Table 3).

**TABLE 2:** The correlations of respiratory support models and laboratory parameters in newborns diagnosed as TTN.

^∗^		Duration of oxygen therapy	Duration of intubation	Duration of nSIMV	Duration of nCPAP	Hospitalization duration
First measured arterial/cord blood pH	*r*	−0.130	0.207	0.016	0.015	0.025
	*p*	**0.019**	0.264	0.863	0.796	0.658

First measured arterial/cord blood pCO_2._ mmHg	*r*	0.188	−0.258	0.027	0.020	−0.031
	*p*	**0.001**	0.161	0.775	0.733	0.575

Calcium, mg/dL	*r*	−0.267	−0.221	−0.192	−0.005	−0.276
	*p*	**< 0.001**	0.233	**0.040**	0.927	**< 0.001**

Phosphorus, mg/dL	*r*	−0.115	0.132	−0.080	0.032	−0.212
	*p*	**0.038**	0.477	0.398	0.573	**< 0.001**

Highest sodium level, mEq/L	*r*	0.159	0.341	0.213	0.071	0.140
	*p*	**0.004**	0.061	**0.023**	0.213	**0.011**

Lowest sodium level, mEq/L	*r*	−0.184	−0.182	−0.065	−0.030	−0.264
	*p*	**0.001**	0.326	0.494	0.595	**< 0.001**

TSH, mU/L	*r*	0.020	0.417	0.201	0.074	−0.082
	*p*	0.773	**0.020**	0.070	0.301	0.235

Hemoglobin, gr/dL	*r*	−0.134	0.079	−0.077	0.018	0.015
	*p*	**0.015**	0.671	0.418	0.749	0.787

Hematocrit, %	*r*	−0.148	0.082	−0.076	−0.015	0.015
	*p*	**0.007**	0.660	0.423	0.788	0.794

MPV, fL	*r*	−0.107	−0.372	−0.033	0.037	−0.134
	*p*	0.053	**0.039**	0.726	0.523	**0.015**

Albumin, gr/dL	*r*	−0.190	−0.237	−0.183	−0.026	−0.271
	*p*	**0.002**	0.243	0.067	0.680	**< 0.001**

*Note:* Statistically significant values are shown in bold.

Abbreviations: MPV, mean platelet volume; nCPAP, nasal continuous positive airway pressure; nSIMV, nasal synchronized intermittent mandatory ventilation; TSH, thyroid‐stimulating hormone.

^∗^Spearman’s correlation test.

**TABLE 3:** Comparison of duration of oxygen therapy with laboratory parameters.

**Oxygen time**	**Unstandardized coefficients**		**Standardized coefficients**			**95% confidence interval for B**	
	**B**	**Standard deviation**	**Beta**	***t***	**Sigma**	**Lower limit**	**Highest limit**

First measured arterial/cord blood PCO_2._ mmHg	0.027	0.012	0.172	2.263	**0.024**	0.003	0.050
Calcium, mg/dL	−0.476	0.169	−0.189	−2.813	**0.005**	−0.809	−0.143
Highest sodium level, mEq/L	0.190	0.037	0.294	5.201	**< 0.001**	0.118	0.262
Lowest sodium level, mEq/L	−0.161	0.031	−0.290	−5.205	**< 0.001**	−0.221	−0.100

*Note:* Linear regression analysis. Statistically significant values are shown in bold.

**FIGURE 1:**
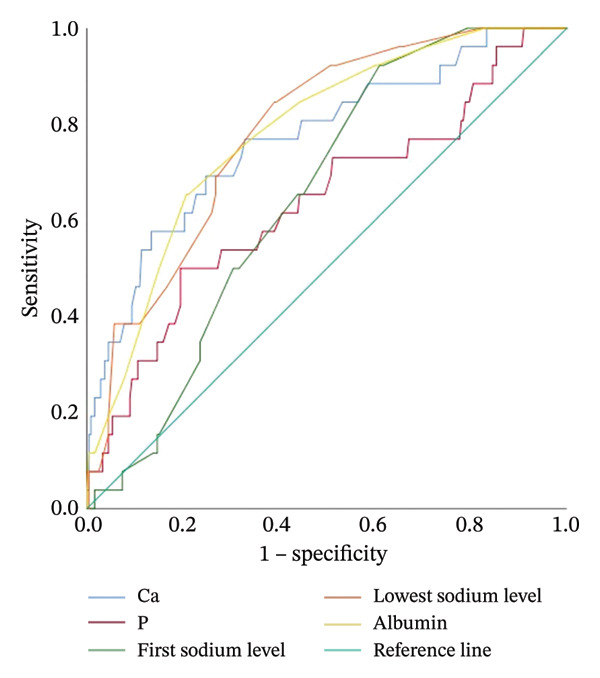
ROC graph showing the effect of calcium, phosphorus, initial sodium, lowest sodium, and albumin values on the need for intubation.

**FIGURE 2:**
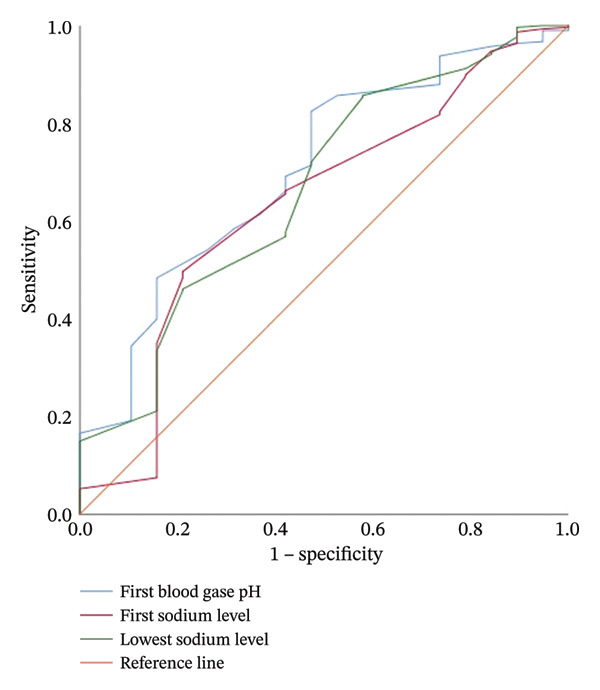
ROC graph showing the effect of the first measured arterial/cord blood pH, initial sodium, and lowest sodium values on the need for nCPAP.

**FIGURE 3:**
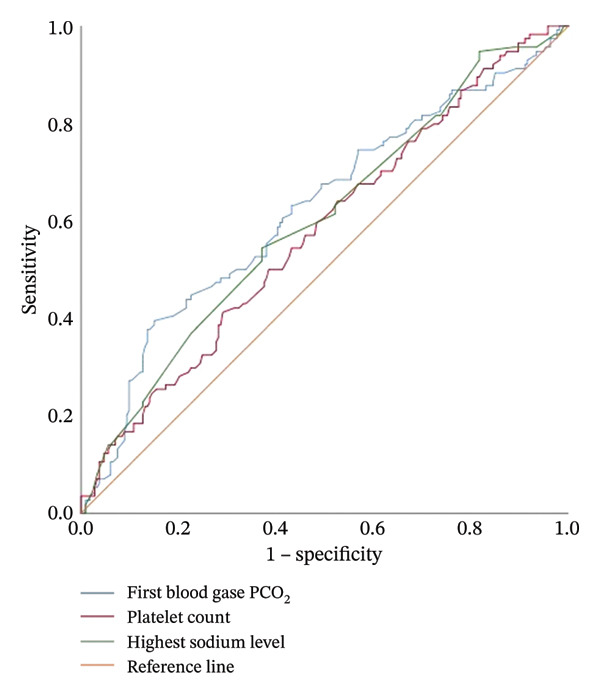
ROC curve showing the effect of pCO2, platelet, and highest sodium values on the need for nSIMV.

In intubated infants, gestational age and birth weight were lower compared to nonintubated infants, while the highest FiO_2_ level and duration of oxygen requirement were higher in intubated infants (Table 4). Laboratory parameters of intubated and nonintubated infants are compared in Table 4. Calcium, phosphorus, initial sodium, and lowest sodium levels, as well as albumin levels, were found to be lower in intubated infants compared to nonintubated infants, while the highest sodium level was higher in intubated infants (Table 4). In ROC analysis used to predict intubation in infants diagnosed with TTN with a gestational age ≥ 35 weeks, the cutoff value for calcium was < 8.11 mg/dL (AUC 0.771, [95% CI: 0.669–0.872], *p* < 0.001), and the cutoff value for phosphorus was < 5.1 mg/dL (AUC 0.637, [95% CI: 0.516–0.759], *p* 0.022). The ability of calcium, phosphorus, albumin, and sodium levels to predict the presence of intubation was examined using ROC analysis and is presented in Table 5 (Figure 1). For predicting need of nCPAP in infants diagnosed with TTN with a gestational age ≥ 35 weeks, the cutoff value for first measured arterial/cord blood pH was < 7.32 (AUC 0.705, [95% CI: 0.586–0.823], *p* 0.003), the cutoff value for PCO_2_ was > 54.5 mmHg (AUC 0.811, [95% CI: 0.701–0.921], *p* 0.002), and the cutoff value for lactate was > 2.66 mmol/L (AUC 0.741, [95% CI: 0.609–0.872], *p* 0.015). In newborns monitored for TTN, the ability to predict the need for nCPAP evaluated by ROC analysis results is presented in Table 5. For predicting need of nSIMV, the cutoff value for first measured arterial/cord blood pH was < 7.28 (AUC 0.599, [95% CI: 0.535–0.663], *p* 0.003), the cutoff value for PCO_2_ was > 62.3 mmHg (AUC 0.623, [95% CI: 0.557–0.688], *p* < 0.001), and the cutoff value for calcium was < 8.27 mg/dL (AUC 0.629, [95% CI: 0.565–0.694], *p* < 0.001). The results of ROC analysis of the ability of laboratory values to predict nCPAP and nSIMV application in infants diagnosed with TTN are presented in Table 5 (Figures 2 and 3).

**TABLE 4:** Comparison of laboratory parameters according to the intubation status of infants monitored in intensive care.

	**Intubation**		**p**
	**No**	**Yes**	
	**Mean ± standard deviation**		

Gender, male, *n* (%)	174 (58.8)	19 (61.3)	0.787
Gestational time (weeks)	37.6 ± 1.6	36.8 ± 1.6	**0.015**
Birth weight, g	3179 ± 539	2977 ± 472	**0.007**
Highest FiO_2_ level, %	27.1 ± 5.1	36.29 ± 14.26	**< 0.001**
Oxygen time, day	1.7 ± 1	5.23 ± 2.87	**< 0.001**
Laboratory values			
First measured arterial/cord blood			
pH	7.24 ± 0.1	7.23 ± 0.1	0.558
pCO_2,_ mmHg	55 ± 11	57.9 ± 10.1	0.138
Lactate, mmol/L	3.3 ± 1.9	2.9 ± 1.5	0.294
Calcium, mg/dL	8.6 ± 0.6	7.9 ±.9	**< 0.001**
Phosphorus, mg/dL	6.1 ±.9	5.5 ± 1.1	**0.006**
First sodium level, mEq/L	138.5 ± 3	137.4 ± 1.8	**0.012**
Highest sodium level, mEq/L	140.9 ± 2.8	142.4 ± 2.7	**0.006**
Lowest sodium level, mEq/L	137.1 ± 3.1	134.0 ± 2.7	**< 0.001**
Hematocrit, %	53.2 ± 6.2	52.9 ± 5.1	0.743
PMI	2421.9 ± 557	2643.5 ± 18.5	0.118
Albumin, gr/dL	3.4 ± 0.3	3.1 ± 0.3	**< 0.001**

*Note:* Statistically significant values are shown in bold.

Abbreviation: PMI: platelet mass index.

**TABLE 5:** Laboratory parameters in infants diagnosed with TTN and the need for intubation, nSIMV, and nCPAP: predicted values obtained from the prediction model and results of significant ROC analysis.

	**Cutoff**	**AUC**	**p**	**95% confidence interval**		**Sensitivity %**	**Specificity %**	**PPV %**	**NPV %**
				**Lowest limit**	**Highest limit**				

*Determining the need for intubation*									
Calcium, mg/dL	< 8.11	**0.771**	**< 0.001**	0.669	0.872	58.1	76.4	20.5	94.6
Phosphorus, mg/dL	< 5.46	**0.637**	**0.022**	0.516	0.759	48.4	80.4	20.6	93.7
First sodium level, mEq/L	< 139.5	**0.651**	**0.011**	0.566	0.736	93.6	38.5	13.7	98.3
Lowest sodium level, mEq/L	< 136.8	**0.785**	**< 0.001**	0.705	0.865	83.9	59.5	17.8	97.2
Highest sodium level, mEq/L	> 140.6	**0.649**	**0.006**	0.550	0.749	74.2	46.6	12.7	94.5
Albumin, gr/dL	< 3.16	**0.782**	**< 0.001**	0.697	0.867	65.4	78.9	24.6	95.6

*Determining the need for CPAP*									
First measured arterial/cord blood									
pH	< 7.32	**0.705**	**0.003**	0.586	0.823	82.5	52.6	96.6	15.6
pCO_2,_ mmHg	> 54.5	**0.811**	**0.002**	0.701	0.921	54.2	79	97.7	9.6
Lactate level, mmol/L	> 2.66	**0.741**	**0.015**	0.609	0.872	60.1	88.9	99.2	9.4
First sodium level, mEq/L	< 138.9	**0.636**	**0.047**	0.501	0.771	49.7	79	97.5	8.8
Lowest sodium level, mEq/L	< 139.7	**0.665**	**0.016**	0.536	0.794	85.7	96	42.1	15.4

*Determining the need for nSIMV*									
First measured arterial/cord blood									
pH	< 7.28	**0.599**	**0.003**	0.535	0.663	78.1	38	40.3	76.4
pCO_2,_ mmHg	> 62.3	**0.623**	**< 0.001**	0.557	0.688	39.5	85	58.4	72.4
Calcium, mg/dL	< 8.27	**0.629**	**< 0.001**	0.565	0.694	46.5	75.1	50	72.4
Phosphorus, mg/dL	< 5.77	**0.569**	**0.040**	0.501	0.637	44.7	70	44.4	70.3
Highest sodium level, mEq/L	> 141.1	**0.600**	**0.003**	0.536	0.664	54.4	62.9	44	72
Lowest sodium level, mEq/L	< 138.6	**0.591**	**0.006**	0.528	0.655	79	34.3	39.1	75.3
Hemoglobin, gr/dL	< 18.35	**0.566**	**0.048**	0.502	0.630	72.8	41.3	39.9	74
Platelet count, 10^9^/L	> 259.5	**0.577**	**0.021**	0.513	0.642	64	47.4	39.5	71.1

*Note:* Statistically significant values are shown in bold.

Abbreviations: AUC, area under the curve; nCPAP, nasal continuous positive airway pressure; NPV, negative predictive value; nSIMV, nasal synchronized intermittent mandatory ventilation; PPV, positive predictive value.

## 7. Discussion

TTN, the most common neonatal respiratory disorder, affects approximately 7%–10% of the neonatal population, and the incidence of new cases is twice that of respiratory distress syndrome [11, 12]. Recently, new information has emerged regarding the pathobiology of TTN. The elimination of alveolar fluid is primarily carried out by two families of molecules. First, epithelial Na + channels (ENaCs), expressed by type‐II alveolocytes and airway epithelial cells, are responsible for Na + uptake across the airway epithelium toward the interstitium; second, aquaporin channels, mainly expressed on type‐I alveolocytes, enable most water passage [2, 13]. Expression of these channels and the Na + ‐K + ‐ATPase pump increases with gestational age. A meta‐analysis reviewing all studies on TTN treatment yielded the following results: *β*2‐Agonists shortened the duration of tachypnea, the time to full feeding, and the length of hospital stay; CPAP reduced the duration of tachypnea; and fluid restriction shortened the length of hospital stay [12]. Currently, there are no enough studies in the literature on the relationship between electrolyte balance and the diagnosis and treatment of TTN. Our study demonstrated that pH and PCO_2_ values in the first blood gas sample after birth, as well as sodium, calcium, and phosphorus levels, can be used to predict the respiratory support treatment model to be applied for infants with a gestational age ≥ 35 weeks diagnosed with TTN.

The importance of respiratory support in TTN for ensuring effective ventilation is well established. It has been reported that oxygen monitoring is mandatory in infants with TTN and that oxygen support should be provided if the saturation level is < 90% [14]. It is also accepted that nCPAP is necessary to increase functional residual capacity in the lungs when respiratory workload increases [2]. It has been reported that blood gas values, particularly partial carbon dioxide pressure, are helpful in assessing the effectiveness of the treatment model in infants requiring respiratory support [2].

The effect of nCPAP treatment administered after birth on TTN prognosis has not been fully demonstrated [15]. In a randomized controlled study of 64 infants with TTN, early nCPAP administration was reported to reduce the duration of tachypnea, the need for intensive care follow‐up, and the need for invasive mechanical ventilation, but did not shorten the length of hospital stay [16]. A meta‐analysis examining the effects of respiratory support applied in TTN treatment showed that nCPAP application significantly reduced the duration of tachypnea symptoms; however, oxygen application, nCPAP, and nSIMV applications did not provide a significant advantage in reducing the frequency of invasive mechanical ventilation requirements [15]. In our study, nCPAP was administered to 94.2% of infants diagnosed with TTN in the delivery room. When comparing the incidence of nCPAP administration in the delivery room with the duration of oxygen requirement, need for intubation, and hospitalization duration in infants followed up due to TTN, no significant difference was found in line with the literature.

In line with the literature, the number of infants receiving noninvasive respiratory support was found to be higher than the number receiving invasive mechanical ventilation support. When infants receiving invasive mechanical ventilation support were evaluated, gestational age and birth weight were lower in those who were intubated compared to those who were not, while the highest FiO_2_ and oxygen duration were found to be significantly higher in intubated infants. There are only a limited number of studies on the predictive value of tests performed during follow‐up in infants diagnosed with TTN. Platelet mass index (PMI) is associated with platelet function and is calculated by multiplying the platelet count by the MPV value. In a retrospective study conducted by Ilhan and colleagues in 2019 on 101 infants with TTN, when all infants were divided into two groups according to their hospitalization duration (less than or greater than 48 h), it was found that the PMI value was significantly lower in the group with a hospitalization duration greater than 48 h, and a cutoff value of 1562 fL/nL was determined [17]. In our study, when PMI indices were compared according to the infants’ nCPAP use, nSIMV use, and intubation status, no statistically significant differences were found between the groups. When the infants’ PMI indices were analyzed with oxygen support duration, nCPAP duration, nSIMV duration, intubation duration, and hospitalization duration, no significant correlations were found. However, when a cutoff value of 1562 fL/nL was used, it was determined that all 21 infants with PMI indices below 1562 fL/nL had hospitalization durations longer than 48 h (average 6 days).

There are very few studies regarding the amount of fluids and electrolyte content to be given to term and late preterm infants diagnosed with TTN. The effect of fluid restriction was evaluated in a prospective randomized controlled trial of 64 late premature and full‐term newborns diagnosed with TTN. Fluid restriction was defined as 60 mL/kg/day on day of life 1 for preterm neonates and 40 mL/kg/day on day of life 1 for term neonates, and total fluid was increased by 20 mL/kg/day daily for all patients until 150 mL/kg/day. No serious adverse effects were reported when moderate fluid restriction was applied [10]. In late premature infants diagnosed with TTN and not exposed antenatal steroid treatment, fluid restriction has been reported to be safe and effective in reducing CPAP duration [18]. However, the meta‐analysis study reported that there is currently insufficient information regarding fluid restriction in the treatment of TTN [4]. We found that low sodium, calcium, and phosphorus levels were shown to be associated with the need for invasive ventilation. The high frequency of intubation in infants with a gestational age ≥ 35 weeks diagnosed with TTN who have electrolyte imbalance highlights the importance of electrolyte monitoring in these infants. It remains unclear whether conditions, such as hyponatremia, hypocalcemia, and hypophosphatemia, are true indicators of disease severity or secondary markers of overall physiological imbalance.

In a prospective study conducted by Elfarargy and colleagues, it was reported that pH levels within the first 24 h after birth had high sensitivity and specificity as cutoff values for predicting TTN development (< 7.36) and respiratory distress syndrome development (< 7.29) [19]. In a retrospective study evaluating 236 infants diagnosed with TTN, it was reported that late preterm birth, umbilical artery blood gas pH < 7.25, and low 1‐min Apgar score were indicators of poor prognosis [20]. The same study reported that the need for mechanical ventilation increased fourfold when blood gas pH was < 7.25 [20]. In our study, when the relationship between pH value and noninvasive respiratory support was examined, it was determined that the need for nCPAP increased when the pH value was < 7.32, and the need for nSIMV increased when the pH value was < 7.28. Our study demonstrated that there is a low but statistically significant association between pH levels and the need for nCPAP and nSIMV.

Özkiraz and colleagues reported in their prospective study conducted in 2013 on 56 infants diagnosed with TTN that lactate levels above 2.5 mmol/L increased the risk of requiring oxygen and mechanical ventilation for more than 72 h in infants [5]. In our study, when analyzing the predictability of nCPAP requirement in infants diagnosed with TTN based on laboratory values, it was found that the rate of nCPAP requirement was higher in infants with lactate levels exceeding 2.66 mmol/L. However, no correlation was found between lactate levels and intubation or invasive mechanical ventilation.

## 8. Limitations

The study was planned as a retrospective and single‐center design so the impact factor of our results is not high. The lack of data on antenatal steroid administration and cord clamping time, which can influence TTN development, is a negative aspect of our study. However, our study included a large number of newborn babies who underwent standard clinical protocol. To improve the clinical reliability of the study, external validation in prospective patient groups is needed.

## 9. Conclusions

In conclusion, our study found that the oxygen usage rate was high in infants with a gestational age ≥ 35 weeks with diagnosed TTN. The pH value in the first blood gas sample taken after birth may be used as a cutoff value to predict the need for nCPAP when the pH value was < 7.32 and the need for nSIMV when the pH value was < 7.28.

A positive and significant correlation was found between PCO_2_ values in cord blood gas and the duration of oxygen requirement. In infants requiring intubation due to TTN, low levels of calcium, phosphorus, and sodium were observed at 24 h. In infants requiring invasive respiratory support, serum sodium levels were found to be higher compared to those who did not require such support.

## Author Contributions

Ali Bulbul, Tolga Bacak, Ahmet Yasar Tellioglu, Alper Divarcı, and Hasan Avsar: investigation, methodology, conceptualization, and writing–original draft. Ali Bulbul and Tolga Bacak: analysis and methodology. Ali Bulbul: project administration, supervision, conceptualization, writing–original draft, and editing.

## Funding

No funding was received for this manuscript.

## Disclosure

All authors read and approved the final manuscript.

## Conflicts of Interest

The authors declare no conflicts of interest.

## Data Availability

The data that support the findings of this study are available from the corresponding author upon reasonable request.
